# Survival Rates and Prognostic Factors in Patients with Coronavirus Disease 2019: A Registry-Based Retrospective Cohort Study

**DOI:** 10.34172/jrhs.2021.47

**Published:** 2021-04-24

**Authors:** Fatemeh Shahbazi, Manoochehr Karami, Mohammad Mirzaei, Younes Mohammadi

**Affiliations:** ^1^Department of Epidemiology, School of Public Health, Hamadan University of Medical Sciences, Hamadan, Iran; ^2^Students Research Committee, Hamadan University of Medical Sciences, Hamadan, Iran; ^3^Modeling of Noncommunicable Diseases Research Center, Hamadan University of Medical Sciences, Hamadan, Iran; ^4^Deputy Minister of Health, Hamadan University of Medical Sciences, Hamadan, Iran; ^5^Social Determinants of Health Research Center, Hamadan University of Medical Sciences, Hamadan, Iran

**Keywords:** COVID-19, Epidemiology, Iran, Mortality, Survival

## Abstract

**Background:** Coronavirus disease 2019 (COVID-19) is a contagious disease caused by a newly identified coronavirus. Our knowledge about the survival rate and prognostic factors of the disease is not established well. Therefore, this study aimed to identify the risk factors associated with the survival of COVID-19 cases in Hamadan province, West of Iran.

**Study design:** A retrospective cohort study

**Methods:** This retrospective cohort study was performed in Hamadan province, West of Iran. The study included patients that referred to the provincial hospitals from February 20 to September 20, 2020. The follow-up of each subject was calculated from the date of onset of respiratory symptoms to the date of death. Demographic and clinical characteristics were extracted from patients’ medical records. Kaplan-Meier method, Flemington-Harrington test, and Cox regression were used for data analysis.

**Results:** The overall 1, 5, 10, 20, 30 and 49-day survival rates were estimated at 99.57%, 95.61%, 91.15%, 87.34%, 86.91%, and 86.74%, respectively. Furthermore, survival time showed a significant association with age, gender, history of traveling to contaminated areas, co-morbidity, neoplasms, chronic diseases, and hospital units.

**Conclusions:** In conclusion, elderly people, male gender, and comorbidities presented a greater risk of death. Therefore, it is important to pay more attention to this group of people to reduce the incidence and consequences after infection.

## Introduction

Coronavirus disease 2019 (COVID-19) is an emerging and major public health problem caused by a newly discovered coronavirus ^1^. By October 2020, the disease had infected more than 35 million cases and killed approximately 1,200,000 people ^2^. Most individuals infected with the COVID-19 virus will experience mild to moderate respiratory illness and attain recovery without any specific treatment ^3^. Older people and those with underlying medical problems, such as cardiovascular disease, diabetes, chronic respiratory disease, and cancer, are more likely to develop serious illness and death ^4-6^. Based on the findings of the previous studies, this disease is associated with complications, such as encephalopathy, thromboembolism, acute myocarditis, rhabdomyolysis, renal failure, heart failure, shock, and multi-organ failure ^6-9^.

Although the clinical and epidemiological aspects of the disease have been widely studied, many other aspects of the disease, including the patient’s survival rate and factors affecting the survival are not well known. The attainment of information about survival rate and effects of risk factors on the survival of the patients is so crucial for policy-makers and health service providers which trades off the existing treatments, assesses drug safety, identifies the factors that increase patient survival, apportions the cost of future medical care, estimates years of life lost, evaluates product reliability, and measures the viability of medical therapies and devices ^10^. Accordingly, this study aimed to specify the survival rate and prognostic factors in patients with COVID-19 in Hamadan province, West of Iran.

## Methods

 This retrospective cohort study was performed from February 20 to September 20, 2020, in Hamadan province, West of Iran. In total, 3,922 patients with positive RT-PCR tests were included in the study using the census method. This study examined all men and women with a confirmed diagnosis of COVID-19 hospitalized in the provincial hospitals. All patients who had undergone anti-COVID-19 treatment were also followed up after discharge up to September 2020. The follow-up of each individual (in person-day) was calculated from the date of onset of respiratory symptoms to date of the death. The patients who survived and the cases who lost follow-up during the study period were considered censored observations.

 The data were collected using a checklist covering such demographic and clinical characteristics as age group (<40, 40-59, ≥60), gender (male, female), place of residence (rural, urban), underlying diseases (yes, no), type of co-morbidity (coronary heart disease [CHD], pulmonary diseases, diabetes, hypertension, neurological disease, neoplasms, liver and kidney diseases, simultaneous infection with several diseases, and other), hospital unit (coronary care unit [CCU], intensive care unit [ICU], general, infectious unit, emergency unit, respiratory isolation section, neonatal care unit, internal care unit), and the history of traveling to contaminated areas (yes, no). The outcome variable was the time from the onset of symptoms (i.e., fever or chills, cough, shortness of breath or difficulty breathing, fatigue, muscle or body aches, headache, new loss of taste or smell, sore throat, congestion, runny nose, nausea or vomiting, and diarrhea) to the occurrence of death.

###  Statistical analysis

 The qualitative data were presented using frequency and percentage, and quantitative data were described as the mean and standard deviation. The survival rate of patients was also compared using Kaplan-Meier survival curves and the Flemington-Harrington test. Furthermore, the descriptive survival information (mean, median, minimum and maximum of survival time) were obtained using the stdes command in Stata software. The survival rates were also calculated by the sts list command which is equivalent to the values displayed in the Kaplan-Meier curves. Finally, a cox-proportional hazard (PH) model or extended-cox model was used to obtain hazard ratio and evaluate the association of survival rates with independent predictors of survival. The Schoenfeld residuals method was utilized to choose the best model (PH cox model or extended cox model). The Schoenfeld residuals model evaluates the PH assumption. If the PH assumption held for all particular covariates, the PH cox model was employed. However, the extended cox model was used if the PH assumption did not hold even for one variable. All statistical analyses were performed in Stata software (version 14; StataCorp, TX, USA), and a p-value less than 0.05 was considered statistically significant.

###  Ethical Consideration

 The study protocol was approved by the Ethics Committee of Hamadan University of Medical Sciences, Hamadan, Iran (IR.UMSHA.REC.1399.633).

## Results

This retrospective cohort study included 3922 patients with a confirmed diagnosis of COVID-19 and a mean age of 56.05 ±19.03 years. Totally, 518 deaths occurred due to COVID-19, and the rest were considered censor observation. More than half of the patients were female (51.38%), and 46.24% of the cases were over 60 years of age; moreover, 73.92% of the individuals lived in urban areas. Comorbidities, such as CHD, diabetes, hypertension, and pulmonary disease were observed in 33.81% of the patients. Furthermore, the majority of corona-positive patients were admitted to the ICU of the hospital (50.23%). The mean mortality rate was 15.3 per 10,000 people (95% CI: 13.89-16.49). This means that for every 10,000 infected person, about 15 of the cases died. As shown in Table 1, the mortality rate from COVID-19 increases with age. Moreover, among all age groups, the highest mortality rate was observed in the age group of 60 years and older. Additionally, the mortality rate of male patients, residents in rural areas, and patients with underlying diseases were higher than females, residents in urban areas, and healthy people. The highest mortality rate was also noted in patients with neoplasms, and the lowest rates were seen in people with hypertension. The minimum and maximum of survival duration lengths among the subjects were estimated at 1 and 230 days, respectively. The last column in Table 1 shows the results of the Flemington-Harington test that evaluates the equivalency of Kaplan-Meier curves in the subgroups of each variable.

**Figure 1:**
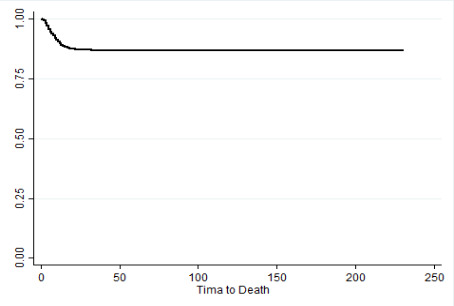
Kaplan-Meier estimate of the surviving function in patients with COVID-19 infection

When the result of this test is statistically significant, it indicates that survival curves are significantly different. More information about patients with COVID-19 is presented in Table 1. Figure 1 illustrates the Kaplan-Meier survival diagram. As it is shown, 1, 5, 10, 20, 30, and 49-day survival probabilities of the patients are obtained at 99.57%, 95.61%, 91.15%, 87.34%, 86.91%, and 86.74%, respectively. Furthermore, the patients who survived more than 49 days after their onset of symptoms had a survival function of the straight line with no reduction in survival probability (Figure 1).

Based on the results in Table 1, there was a significant difference in the proportion of COVID-19 positive subjects who progressed to death regarding age group, gender, underlying diseases, comorbidity, hospital units (*P*=0.001 for all these variable), and history of traveling to contaminated areas (*P*=0.028). However, there was no statistically significant difference between rural and urban areas regarding the proportion of progression from COVID-19 infection to death (*P*=0.236).

**Table 1:** Clinical and demographical characteristics of the patients with COVID-19 infection and result of Flemington-Harrington test

**Variables**	**Number (%)**	**Mortality rate** **per 10,000 person**	**Survival time (Days)**		
			**Mean**	**Median**	***P*** **-value**
Age groups (yr)					0.001
<40	912 (23.26)	2.95 (2.02, 4.30)	100.3	89.0	
40-59	1196 (30.50)	6.62 (5.28, 8.30)	94.7	87.0	
≥60	1813 (46.24)	30.30 (27.52, 33.36)	75.7	71.0	
Gender					0.001
Female	2015 (51.38)	11.97 (10.48, 13.69)	89.1	82.0	
Male	1907 (48.62)	18.61 (16.63, 20.83)	85.4	79.0	
Place of residence					0.326
Rural	1023 (26.08)	16.14 (13.71, 19.01)	87.2	79.0	
Urban	2899 (73.92)	14.78 (13.35, 16.35)	87.3	80.0	
History of traveling to contaminated areas				0.028
No	3863 (98.50)	15.49 (14.21, 16.88)	86.3	79.0	
Yes	59 (1.50)	2.20 (0.55, 8.80)	154.2	181.0	
Underlying diseases					0.001
No	2596 (66.19)	8.26 (7.19, 9.48)	94.2	86.5	
Yes	1326 (33.81)	32.33 (28.96, 36.10)	73.8	70.0	
Type of diseases					0.001
Coronary heart disease	548 (41.05)	37.19 (31.29, 44.19)	63.3	65.5	
Pulmonary diseases	131 (9.81)	33.07 (23.51, 46.52)	76.2	72.0	
Diabetes	286 (21.42)	21.15 (15.94, 28.06)	79.6	75.0	
Hypertension	48 (3.60)	13.29 (6.92, 25.55)	141.1	175.0	
Neurological disease	43 (3.22)	27.66 (14.88, 51.41)	84.1	71.0	
Neoplasms	38 (2.85)	110.007 (71.01, 170.61)	47.8	15.0	
Liver and kidney diseases	47 (3.52)	40.96 (24.26, 69.16)	72.7	71.0	
Simultaneous infection	118 (8.84)	55.01 (40.93, 73.92)	67.8	53.0	
Other	76 (5.69)	16.49 (9.36, 29.04)	95.7	93.5	
Hospital units					0.001
Cardiac Care Unit	61 (1.56)	54.36 (35.07, 84.26)	60.3	54.0	
Intensive Care Unit	1970 (50.23)	19.09 (16.97, 21.47)	73.9	71.0	
General	444 (11.32)	16.11 (12.21, 21.25)	69.9	77.0	
Infectious	107 (2.73)	4.65 (2.32, 9.29)	160.9	177.0	
Emergency	65 (1.66)	28.53 (17.48, 46.57)	86.3	88.0	
Respiratory isolation unit	786 (20.04)	12.06 (9.91, 14.67)	105.5	97.5	
Neonatal care unit	39 (0.99)	3.29 (0.46, 23.38)	77.8	70.0	
Internal care unit	375 (9.56)	9.81 (7.22, 13.2)	111.4	100.0	
Other	75 (1.91)	3.52 (1.32, 9.37)	151.6	169.0	

**Figure 2:**
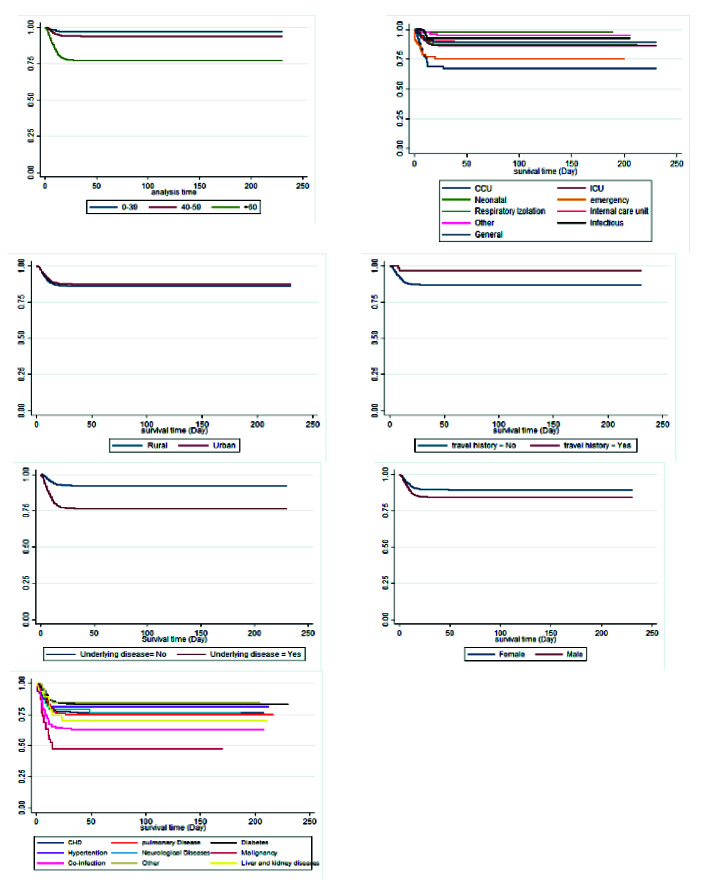
Kaplan-Meier estimate of the surviving function for all independent variables

Table 2 tabulates the results of the univariate and multivariable analysis using the cox PH model. The second column in Table 2 shows the p-value for the Schoenfeld residual. Schoenfeld residuals examined the PH assumption. The p-values are quite high for all variables, suggesting that all variables satisfy the PH assumption; accordingly, the PH cox model, not the extended cox model, was employed in this study. Based on the univariate and multivariable analyses, the male gender, age over 60 years, and co-infection with multiple diseases are associated with a statistically significant increased risk of death among COVID-19 patients. Figure 2 illustrates the Kaplan-Meier function of the variables related to the patients' survival based on the results in Table 2.

## Discussion

 This study aimed to specify the effect of prognostic factors on the survival of COVID-19 patients using a PH cox model. Based on the obtained results, the overall 1, 5, 10, 20, 30 and 49-day survival rates were 99.57%, 95.61%, 91.15%, 87.34%, 86.91%, and 86.74%, respectively. In addition, survival time showed a significant association with age, gender, history of traveling to contaminated areas (the contaminated areas are the provinces of the country that were in the red situation by the ministry of health and medical education, and the person had traveled to those areas a week before the onset of symptoms), underlying diseases, neoplasms, chronic diseases, and hospitalization sector.

The present study indicated that elderly patients with COVID-19 had the highest mortality rate and lowest survival rate. This finding is consistent with the results of previous studies which demonstrated a higher mortality rate among the elderly populations ^11,12^. Principally, elderly people have a weak immune response to infectious agents, and therefore, are more susceptible to severe infection ^13^. On the other hand, the prevalence of bacterial infection and underlying diseases, such as diabetes, hypertension, cardiovascular disease, and cerebrovascular disease, is higher in the elder population, compared to young and middle-aged patients. This puts them at a higher risk of COVID-19 infection and its adverse consequences, including death. Additionally, in Severe Acute Respiratory Syndrome (SARS) and Middle East Respiratory Syndrome (MERS) diseases, aging has been introduced as an important independent risk factor for mortality ^14^.

Our findings showed that the median and mean survival time is significantly lower in males, compared to females. Epidemiological studies show gender-specific differences in the incidence and mortality rates in humans after COVID-19 infection with males experiencing a higher mortality rate, compared to females ^15^. Previous investigations also revealed that men manifested more serious forms of the disease during the COVID-19 epidemic, compared to women ^15-17^. This decreased vulnerability of women to viral infections may be attributed to the sex hormones and the X chromosome, which perform an essential role in innate and adaptive immunity ^18^. On another aspect, a higher incidence rate of COVID-19 in men might be due to higher social interactions at workplaces. National office for statistics reported that men included 81% of the workforce in Iran during 2018-19, while more than 50% of them were employed in service occupations. Therefore, there is a higher possibility for men to obtain COVID-19 infection due to higher social interactions in work environments ^19^.

**Table 2:** Result of univariate and multivariate Cox proportional hazards survival analysis in COVID-19 patients

**Variables**	***P*** **-value for** **Schoenfeld residuals**	**Crude HR** **(95% CI)**	***P*** **-value**	**Adjusted** **HR (95% CI)**	***P*** **-value**
Age groups (year)	0.192				
<40		1.00		1.00	
40 to 59		2.15 (1.38, 3.33)	0.001	0.87 (0.45, 1.69)	0.683
≥60		8.58 (5.81, 12.67)	0.001	2.56 (1.41, 4.62)	0.002
Gender	0.497				
Female		1.00		1.00	
Male		1.53 (1.28, 1.82)	0.001	1.74 (1.38, 2.18)	0.001
Place of residence	0.799				
Rural		1.00		1.00	
Urban		0.91 (0.75, 1.11)	0.28	0.90 (0.70, 1.15)	0.390
History of traveling to contaminated areas	0.781				
No		1.00		1.00	
Yes		0.91 (0.06, 0.96)	0.044	0.51 (0.12, 2.06)	0.344
Underlying diseases	0.428				
No		1.00		1.00	
Yes		3.36 (2.81, 4.01)	0.001	0.64 (0.20, 2.04)	0.456
Type of diseases	0.309				
Hypertension		1.00		1.00	
Pulmonary diseases		1.28 (0.65, 2.51)	0.474	1.44 (0.69, 2.98)	0.326
Diabetes		1.34 (0.64, 2.81)	0.430	1.61 (0.73, 3.52)	0.235
Coronary heart disease		0.86 (0.42, 1.76)	0.690	1.15 (0.54, 2.44)	0.724
Neurological disease		1.26 (0.51, 3.11)	0.610	1.98 (0.77, 5.06)	0.155
Neoplasms		3.48 (1.58, 7.65)	0.002	4.53 (1.97, 10.41)	0.001
Liver and kidney diseases		1.60 (0.69, 3.69)	0.274	2.18 (0.90, 5.26)	0.084
Simultaneous infection		2.23 (1.08, 4.56)	0.029	2.74 (1.29, 5.79)	0.008
Other		0.81 (0.34, 1.91)	0.627	1.28 (0.52, 3.17)	0.588
Hospital units	0.865				
Neonatal care unit		1.00		1.00	
Cardiac Care Unit		15.09 (2.03, 112.49)	0.008	2.76 (0.34, 22.60)	0.344
Intensive Care Unit		5.79 (0.81, 41.22)	0.080	1.59 (0.21, 12.46)	0.661
General		4.55 (0.62, 32.92)	0.134	1.55 (0.19, 12.40)	0.676
Infectious		2.91 (0.36, 23.29)	0.313	1.99 (0.22, 17.48)	0.539
Emergency		11.32 (1.50, 85.39)	0.019	4.91 (0.57, 42.06)	0.147
Respiratory isolation unit		5.16 (0.72, 36.97	0.103	1.78 (0.22, 14.09)	0.587
Internal care unit		4.39 (0.60, 31.97)	0.143	2.46 (0.30, 19.87)	0.398
Others		2.07 (0.23, 18.52)	0.515	0.90 (0.08, 10.49)	0.930

Our findings revealed that the mortality rate of COVID-19 in the residents of rural areas was higher than that in urban areas; however, their survival function was not significantly different. The high mortality rate in rural areas may happen because of factors correlated with poor access to healthcare or inadequate surveillance and monitoring in rural regions ^20^.

According to the present study, the mortality rate of COVID-19 in patients with underlying diseases is four times higher than that in healthy people. On the other hand, survival time in people with the underlying diseases is significantly shorter than that in people who do not have these diseases. Previous literature showed that underlying diseases, such as diabetes, hypertension, and coronary heart disease, increased the risk of COVID-19 infection and subsequent adverse consequences, such as hospitalization in ICU and death ^4,21,22^. This occurs because of several mechanisms, including direct damage by the virus, systematic inflammatory responses, and weakened immune system. According to a study conducted by Emami et al., patients with neoplasms were more in danger for mortality from COVID-19 than those without any tumor, which was consistent with the results of the present study ^4.^ Anticancer treatments, such as chemotherapy and surgery, put this group into an immunosuppressive state and subsequently at higher risk of MERS-CoV-2 infection ^23^.

 There were some limitations in our study. First, estimation of survival rate requires reliable sources of data obtained from the prospective design; however, this study was conducted based on a retrospective cohort design. Second, information about potential confounding factors was not available, such as access to health care insurance and the severity of the disease. Moreover, this study was performed in a specific geographic area of Iran. On the other hand, there might be some unknown genetic or environmental factors influencing the results; therefore, the findings might not be completely generalizable to other populations. Despite these limitations, the authors were able to use the estimated 20 and 49-day survival rates measuring the time from symptom onset to outcome.

## Conclusions

 In conclusion, our findings demonstrated that several factors, such as age (elderly population), male gender, as well as simultaneous infection and neoplasms, increased the risk of mortality from COVID-19 infection. Infection prevention and control strategy plan include entry/exit screening, restriction of movement, closure education centers, wearing the mask, imposing quarantine, and active surveillance.

## Acknowledgment

 This study (ID: IR.UMSHA.REC.1399.633) was funded by the Deputy of Research and Technology of Hamadan University of Medical Sciences, Hamadan, Iran. The funder had no role in study design, data collection, analysis, decision to publish, or preparation of the manuscript.

## Conflict of interest

 The authors have declared no conflicts of interest.

## Funding

 The study was funded by the Hamadan University of Medical Sciences, Hamadan, Iran (No. 9910237363). Funder has no role in the design of the study, data collection, analysis, interpretation of data, and writing the manuscript.

## Authors’ contributions:

 All authors gave final approval of the version to be published and agreed to be accountable for all aspects of the work in ensuring that questions related to the accuracy or integrity of any part of the work were appropriately investigated and resolved. FSH: Conceptualization, Methodology, Data curation, Formal analysis, Writing-Original draft, and final manuscript. YM: Conceptualization, Methodology, Supervision of the paper writing. MK: Methodology, Supervision of the paper writing. MM: Data preparation.

## Ethics approval

 The study protocol was approved by the Ethics Committee of Hamadan University of Medical Sciences, Hamadan, Iran (IR.UMSHA.REC.1399.633).

## Highlights

- The mean mortality rate from COVID-19 was 15.3 per 10,000 people (95% CI: 13.89, 16.49)
- The overall 1, 5, 10, 20, 30 and 49-day survival rates were estimated at 99.57%, 95.61%, 91.15%, 87.34%, 86.91%, and 86.74%, respectively.
- The minimum and maximum survival times in the subjects were 1 and 230 days, respectively.
- Survival time showed a significant association with age, gender, history of traveling to contaminated areas, co-morbidity, neoplasms, chronic diseases, and hospital units.
